# Courage Unmasked

**DOI:** 10.3201/eid2005.AC2005

**Published:** 2014-05

**Authors:** Sharon Bloom

**Affiliations:** Centers for Disease Control and Prevention, Atlanta, Georgia, USA

**Keywords:** head and neck cancer, oropharyngeal cancer, human papillomavirus, HPV, radiation mask, flower mask, about the cover, Courage Unmasked, With Apologies to Arcimboldo, B.J. Adams, emerging infectious diseases, art and medicine

**Figure:**
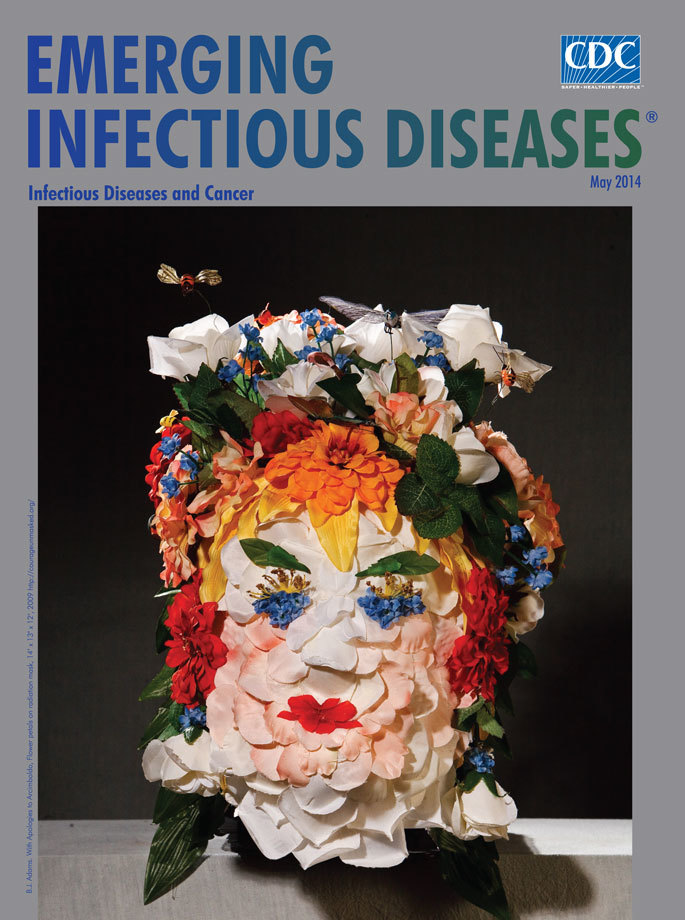
B.J. Adams. *With Apologies to Arcimboldo* (detail), 2009. Flower petals on radiation mask, 14″ x 13″ x 12″. 9114hnc.org

Lying supine, the technician uses my mask to bolt me to the treatment table.

After he leaves the room, I feel alone, and at first, afraid.

I see and hear the radiation machine move around me, but feel nothing.

Minutes later, the door opens, and the mask is unbolted.

This process is repeated almost daily for 2 months…

After 2 weeks, the skin on my neck and inside my mouth becomes red, sore, and dry.

My sense of taste changes, it becomes difficult to eat.

By the end, the technician has become my trusted friend,

I can not predict the future, but I feel less afraid, my

Courage Unmasked.

—Cookie Kerxton (pers. comm., February 2014)

Radiation treatment for head and neck cancer involves the use of custom-molded plastic mesh masks placed over the head and shoulders to immobilize the patient and facilitate the stereotactic positioning of radiation beams. This month’s cover art was selected from the “Courage Unmasked” project, which was founded by Cookie Kerxton, a head and neck cancer survivor and artist, who during one such treatment session envisioned the use of art to both raise awareness of and support fellow patients in need. After the discarded masks have been transformed into works of art, they are exhibited and auctioned off.

Fiber artist B.J. Adams was one of more than 100 artists invited to create art from used radiation masks with materials of their choice. Inspired by Italian artist Giuseppe Arcimboldo (1527–1593), who created portraits by using objects, such as flowers, vegetables, and fruit, Adams chose brightly colored artificial flowers and insects to create a spring-like mask to symbolize rebirth and rejuvenation. Adams was born in California and studied fine art; she has been working in mixed media for ≈60 years and lives in Washington, DC.

In some countries, human papillomavirus (HPV) is found in most oropharyngeal cancers, a subset of head and neck cancers. New data from the United States show that the association of HPV with oropharyngeal cancers is more common than previously believed; furthermore, the most common HPV types detected in these cancers are types prevented by available vaccines. It is thus conceivable that HPV vaccines developed to prevent cervical cancer might also prevent some oropharyngeal cancers.
